# Dissecting the Genetic Architecture of Seed Protein and Oil Content in Soybean from the Yangtze and Huaihe River Valleys Using Multi-Locus Genome-Wide Association Studies

**DOI:** 10.3390/ijms20123041

**Published:** 2019-06-21

**Authors:** Shuguang Li, Haifeng Xu, Jiayin Yang, Tuanjie Zhao

**Affiliations:** 1 Huaiyin Institute of Agricultural Sciences of Xuhuai Region in Jiangsu/Huai’an Key Laboratory for Agricultural Biotechnology, Huai’an 223001, China; dawn0524@126.com (S.L.); hanksxhf@163.com (H.X.); 2 Soybean Research Institution, National Center for Soybean Improvement, Key Laboratory of Biology and Genetics and Breeding for Soybean, Ministry of Agriculture, State Key Laboratory of Crop Genetics and Germplasm Enhancement, Nanjing Agricultural University, Nanjing 210095, China

**Keywords:** soybean, genome-wide association study, seed protein content, seed oil content

## Abstract

Soybean is a globally important legume crop that provides a primary source of high-quality vegetable protein and oil. Seed protein and oil content are two valuable quality traits controlled by multiple genes in soybean. In this study, the restricted two-stage multi-locus genome-wide association analysis (RTM-GWAS) procedure was performed to dissect the genetic architecture of seed protein and oil content in a diverse panel of 279 soybean accessions from the Yangtze and Huaihe River Valleys in China. We identified 26 quantitative trait loci (QTLs) for seed protein content and 23 for seed oil content, including five associated with both traits. Among these, 39 QTLs corresponded to previously reported QTLs, whereas 10 loci were novel. As reported previously, the QTL on chromosome 20 was associated with both seed protein and oil content. This QTL exhibited opposing effects on these traits and contributed the most to phenotype variation. From the detected QTLs, 55 and 51 candidate genes were identified for seed protein and oil content, respectively. Among these genes, eight may be promising candidate genes for improving soybean nutritional quality. These results will facilitate marker-assisted selective breeding for soybean protein and oil content traits.

## 1. Introduction

Soybean (*Glycine max* (L.) Merr.) is a globally important legume crop, the seed of which contains approximately 40% protein and 20% oil, accounting for 70% of world protein meal and 29% of vegetable oil consumption [1]. Soybean provides a great source of human food, cooking oil, and livestock feed, and has biodiesel production, industrial, and pharmaceutical applications [2]. The phenotypic range of protein content of soybean has been reported to be 34.1–56.8% of seed dry mass, and oil content ranged from 8.3 to 27.9% [3], suggesting that there is great potential for genetic improvement of soybean seed protein and oil content. Breeding cultivated soybean varieties with high-protein and high-oil is an extremely important and promising objective. The negative correlation between protein and oil content make improvement of both traits simultaneously a challenging task using conventional breeding [4]. Therefore, the identification of molecular markers associated with quantitative trait loci (QTLs) controlling protein and oil content is a necessary prerequisite for breaking the negative correlations between both the traits [5].

Seed protein and oil content of soybean are quantitatively inherited and governed by multiple genetic loci subject to genotype × environment interactions [6,7,8]. Given the great agricultural importance of protein and oil content in soybean, many QTLs controlling these two seed traits have been reported over the past two decades. Prior to this study, there were 241 QTLs for protein content and 315 QTLs for oil content in the soybean genetics and genomics database (SoyBase, https://soybase.org/), distributed over 20 soybean chromosomes. The majority of these QTLs were mapped by linkage mapping based on biparental populations such as the recombinant inbred line (RIL), F2, and backcross populations. However, these linkage mapping studies were limited by the relatively small phenotypic variation of biparental populations and by the fact that only two alleles per locus can be studied simultaneously. The broad chromosome regions of QTLs make it especially difficult to identify putative candidate genes of interest [9,10,11].

Association mapping based on natural populations offers higher mapping resolution, enables examination of a greater number of alleles, and requires less time for establishing populations compared to linkage mapping [12]. Currently, genome-wide association study (GWAS) with high-throughput single nucleotide polymorphism (SNP) genotyping has emerged as a promising procedure for dissecting the genetic architecture of agronomic, seed composition traits in soybean [13,14,15]. Patil et al. [8] reviewed the progress in molecular mapping and genomics of soybean seed protein, and concluded that major QTLs for soybean protein were detected repeatedly on chromosomes 20 and 15. Using GWAS, Bandillo et al. [16] identified 19 and 18 significantly associated SNPs for protein and oil content, respectively, and found clusters of strong association signals on chromosomes 20 and 15 using 12,116 *G. max* accessions from the United States Department of Agriculture (USDA) soybean germplasm collection. Zhang et al. [17] and Zhang et al. [18] identified 89 QTLs for seed protein content and 50 QTLs for seed oil content and annotated putative candidate genes using 365 accessions from the Chinese soybean landrace population. Li et al. [7] identified 14 SNPs correlated with protein content and 12 SNPs correlated with oil content in a population of 185 soybean germplasms. These studies confirmed QTLs previously identified by linkage mapping and detected many novel QTLs; they also indicated that both seed protein and oil content in soybean are complex quantitative traits controlled by multiple genes. Further surveys are needed to obtain a comprehensive understanding of the genetic architecture of seed protein and oil content by applying more efficient association mapping procedures. Particularly, multi-locus GWAS procedures, which do not require multiple test correction due to their multi-locus nature and which improve the power and accuracy of QTL detection, have served as excellent mapping procedures [19,20,21]. Recently, He et al. [22] proposed an innovative restricted two-stage multi-locus multi-allele GWAS (RTM-GWAS) procedure for a relatively thorough detection of QTLs and the multi-allelic variation which widely exists in germplasm populations. The RTM-GWAS has two features that distinguish it from other multi-locus procedures: the tightly linked SNPs are divided into SNP linkage disequilibrium blocks (SNPLDBs) to construct genomic markers with multiple haplotypes as alleles, and then two-stage association analysis is performed using the multi-locus multi-allele model. The RTM-GWAS procedure has been utilized for QTL detection of 100-seed weight [23], seed isoflavone content [24], seed protein content [17], and seed oil, oleic acid, and linolenic acid content [18] in the Chinese soybean landrace population. It has also been used for QTL detection of flowering date [25] and drought tolerance at the seedling stage [26] in a nested association mapping population, and flowering date of an RIL population [27] in soybean.

In the present study, we utilized the RTM-GWAS procedure to dissect the genetic architecture of seed protein and oil content in an association panel of 279 soybean accessions which was collected from the Yangtze and Huaihe River Valleys in China, an important summer sowing soybean production area, and the association panel was genotyped with 59,845 SNP markers using restriction-site-associated DNA sequencing. The objectives of this study were to identify QTLs and their multiple alleles for seed protein and oil content and to deduce potential candidate genes located within associated genomic regions in order to facilitate the genetic improvement of seed protein and oil content obtained through soybean molecular breeding programs.

## 2. Results

### 2.1. Phenotypic Variation of Seed Protein and Oil Content

Table 1 shows that the seed protein and oil content varied widely in the association panel. Seed protein content ranged from 37.2% to 47.6% with an average of 42.6% in 2015, and from 36.1% to 49.3% with an average of 42.6% in 2016 (Figure S1A). Seed oil content ranged from 15.2% to 21.4% with an average of 18.5% in 2015, and from 14.8% to 21.1% with an average of 18.2% in 2016 (Figure S1B). A highly significant negative correlation was observed between protein and oil content (*r* = −0.93; *p*-value < 0.0001) (Figure S2). The broad-sense heritabilities were estimated as 80.4% and 79.0% for protein and oil content, respectively. The analysis of variance (ANOVA) results (Table 1) showed significant differences across accessions, and a significant accession × environment interaction for both protein and oil content.

### 2.2. Genetic Diversity and Population Structure Analysis Based on SNPLDB Markers

Among the 279 soybean accessions of the association panel, 59,845 SNPs with minor allele frequency (MAF) ≥5% were identified by restriction site-associated DNA sequencing (RAD-seq) and organized into 8148 SNPLDBs, of which 4402 SNPLDBs comprised multiple SNPs, while the remaining 3748 SNPLDBs comprised only a single SNP. There were 2–12 alleles with an average of 3.1 alleles for each SNPLDB, and the average MAF significantly decreased to 0.115 due to the increase in the number of allele per marker, as compared with the average MAF (0.217) of SNP markers having only two alleles [28]. The polymorphic information content (PIC) for each locus ranged from 0.091 to 0.842, with an average of 0.351.

**Figure 1:**
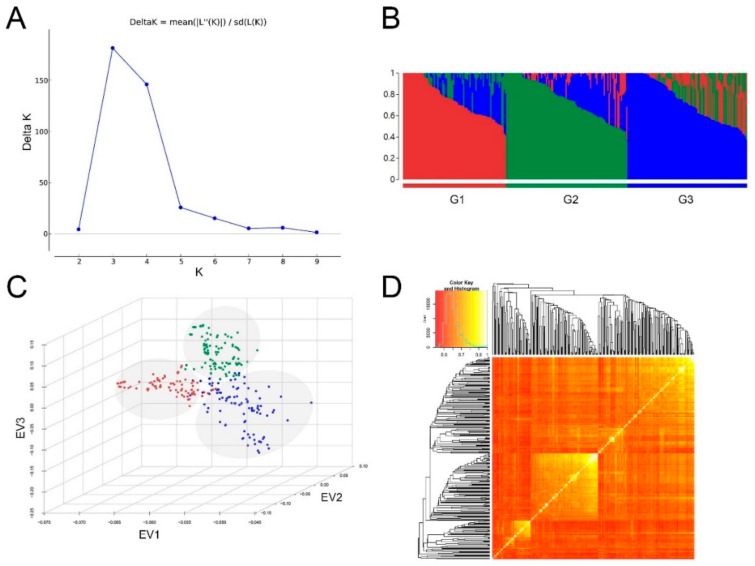
Population structure analysis of the soybean association panel using 8148 single nucleotide polymorphisim linkage disequilibrium blocks (SNPLDB) markers. (**A**) Estimation of the number of subpopulations using the STRUCTURE program. Delta K shows K = 3 as the most likely number of subpopulations. (**B**) Population structure bar plot at K = 3 inferred by the STRUCTURE program. Each bar represents one accession, and the bars are filled by colors representing the likelihood of membership to each subpopulation. G1 (red), G2 (green), and G3 (blue) denote three subpopulations. (**C**) Scatter plot of the top three eigenvectors (EV1, EV2, and EV3) with the largest eigenvalues of the genetic similarity coefficient matrix based on SNPLDB markers, which were calculated by restricted two-stage multi-locus genome-wide association analysis (RTM−GWAS) software. Each dot in the scatterplot represents an accession. Accessions are colored according to the subpopulation they were assigned to by STRUCTURE at K = 3. (**D**) Heatmap of genetic similarity coefficient matrix based on SNPLDB markers.

A previous study suggested that there were three distinct subpopulations in this association panel based on SNP markers [28]. Similarly, in this study, the most likely value of K was 3 based on delta K information from the STRUCTURE analysis using SNPLDB markers (Figure 1A), suggesting that the association panel could be partitioned into three genetically distinct subpopulations (Figure 1B). This result is also in accordance with eigenvector analysis (Figure 1C), and a heatmap of the genetic similarity coefficient (GSC) matrix based on SNPLDB markers (Figure 1D).

### 2.3. Genome-Wide Association Study for Seed Protein and Oil Content

In this study, 279 soybean accessions with 8148 SNPLDBs were used to perform association study using the multi-locus multi-allele RTM-GWAS procedure developed by He et al. [22]. The top 10 eigenvectors with the largest eigenvalues from the GSC matrix calculated based on genome-wide SNPLDBs were incorporated as covariates for population structure correction.

**Figure 2:**
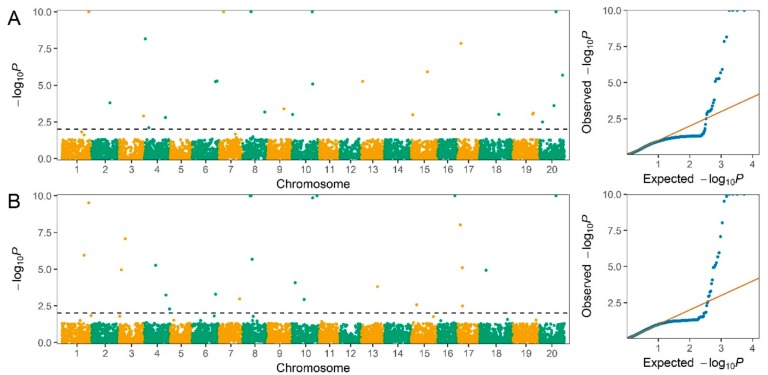
Manhattan and quantile-quantile plots illustrating GWAS for seed protein content (**A**) and oil content (**B**) based on SNPLDB markers using the RTM-GWAS procedure in the soybean association panel. The horizontal dotted black line indicates the genome-wise significance threshold of 0.01, where the −log *P* values of SNPLDB markers for seed protein content and oil content range from 2.1 to 56.1 (A) and 2.3 to 55.0 (B), respectively. The −log *p* values greater than 10.0 are shown as 10.0.

For seed protein content, 26 QTLs distributed on 15 chromosomes (1, 2, 3, 4, 6, 7, 8, 9, 10, 13, 15, 17, 18, 19, 20) were detected, and the −log *P* values of these QTLs ranged from 2.1 to 56.1. The genetic contribution per QTL (*R^2^*) ranged from 0.4% to 16.0%, with a sum of 58.3% of the total phenotypic variance (Figure 2A and Table 2). Of the detected QTLs, 16 large-contribution QTLs (*R^2^* ≥ 1%) collectively explained 51.4% of phenotypic variation, while 10 small-contribution QTLs (*R^2^* < 1%) explained a total of 6.9% of phenotypic variation. The QTL *qProt-20-3* located on chromosome 20 explained 16.0% of the phenotypic variation, providing the largest genetic contribution of the detected QTLs for seed protein content, with the second and third largest genetic contributions made by *qProt-8-1* at 8.0% and *qProt-7-1* at 4.7%, respectively.

For seed oil content, 23 QTLs located on 13 chromosomes (1, 3, 4, 6, 7, 8, 10, 13, 15, 16, 17, 18, 20) were identified, and their −log *P* values varied from 2.3 to 55.0. The genetic contribution of per QTL (*R^2^*) varied from 0.4% to 15.1%, collectively accounting for 53.1% of the phenotypic variance (Figure 2B and Table 3). Among the detected QTLs, 17 large-contribution QTLs (*R^2^* ≥ 1%) collectively explained 49.6% of phenotypic variation, while six small-contribution QTLs (*R^2^* < 1%) explained a total of 3.4% of phenotypic variation. The QTL *qOil-20-1* on chromosome 20 explained 15.1% of the phenotypic variation, which had the largest genetic contribution of the identified QTLs for seed oil content, with the second and third largest genetic contributions made by *qProt-8-2* at 5.2% and *qOil-10-4* at 4.6%, respectively.

### 2.4. QTL-Allele Matrices of Seed Protein and Oil Content

**Figure 3:**
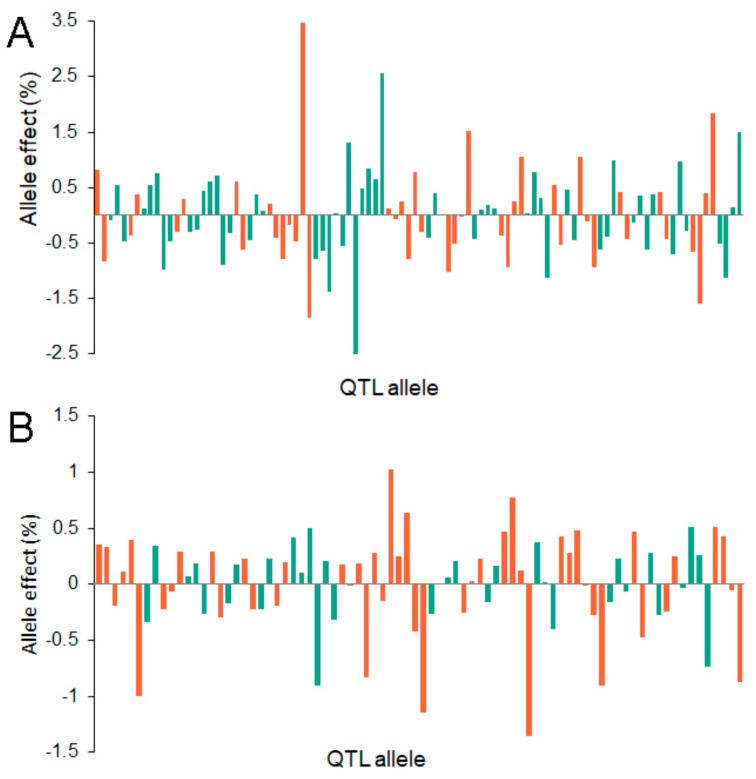
The allele effects of the QTLs associated with the seed protein (**A**) and oil (**B**) content detected in the soybean association panel. The red and green bars which take turns represent the different QTLs, respectively. The bars above the abscissas represent positive values while the bars below the abscissas represent negative values. There are 26 QTLs with 98 alleles for seed protein content (**A**) and 23 QTLs with 80 alleles for seed protein oil content (**B**), respectively.

There were 98 alleles on 26 QTLs for seed protein content and 80 alleles on 23 QTLs for oil content, and their allele effects were estimated using the RTM-GWAS procedure. The number of alleles for each QTL ranged from 2 to 11 for both traits, and the QTLs *qProt-8-1* and *qOil-8-2* had the largest number of alleles. There were 48 and 45 positive (increased protein or oil content) and 50 and 35 negative (decreased protein or oil content) alleles for seed protein and oil content, respectively. The allelic effect sizes ranged from −2.50% to 3.47% for seed protein content, and −1.36% to 1.02% for seed oil content, while the majority of the allele effects varied between −1% and 1% (Figure 3A).

**Figure 4:**
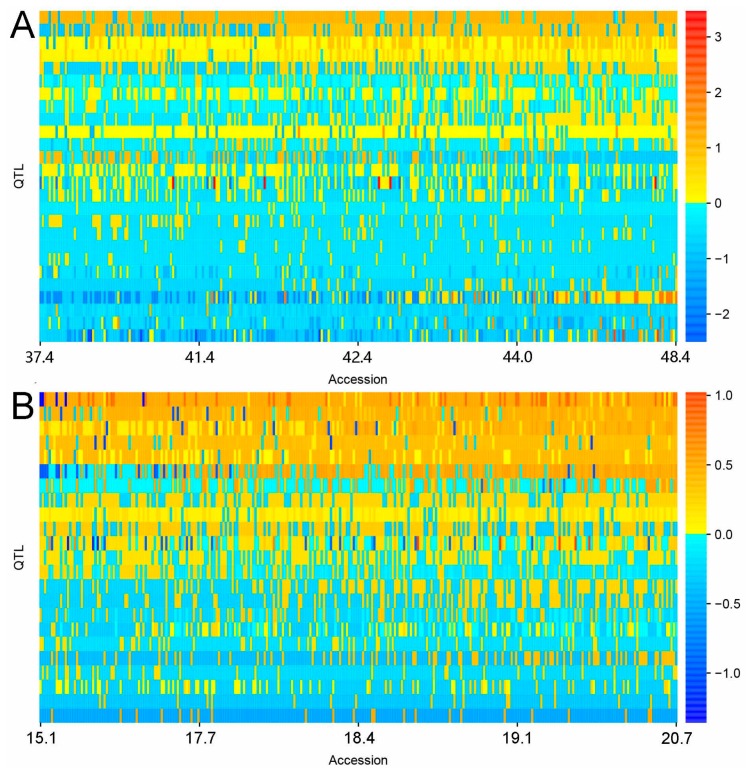
Graphical representation of QTL–allele matrices of seed protein (**A**) and oil (**B**) content detected in the soybean association panel. The horizontal axis indicates accessions arranged in ascending order of seed protein or oil content (%), while the vertical axis indicates QTLs arranged in ascending order of their positive allele frequency. Every row indicates the allele distribution among accessions at a QTL, while every column indicates the allele constitution of an accession over all QTLs. Allele effects are expressed in color cells where warm colors indicate positive effects, cool colors indicate negative effects, and the color gradient indicates effect size.

As shown in Figure 4, the allele effects of the detected QTLs in the 279 soybean accessions were organized into 26 × 279 and 23 × 279 (QTL × accession) QTL–allele matrices for seed protein and oil content of the soybean association panel, respectively. These QTL–allele matrices encompassed the genetic constitution of the population, including QTLs and corresponding allele effects and frequencies. No accession contained all negative or all positive alleles on all detected QTLs for either seed protein or oil content, and the accessions of high protein content (or oil content) had more positive alleles than those of low protein content (or oil content). The difference between high and low protein content (or oil content) accessions resulted from the different compositions of the positive and negative alleles. Most notably, there were many positive alleles in low protein content (or oil content) accessions and negative alleles in high protein content (or oil content) accessions, suggesting that seed protein and oil content possessed large recombination potential in the association panel.

### 2.5. The Common QTLs Associated with Seed Protein and Oil Content

Out of the detected QTLs, five pairs of common QTLs located on chromosomes 1, 6, 8, 10 and 20 were associated with both seed protein and oil content (Table 4), indicating these loci had possible pleiotropic effects or very tight linkage, leading to a significant negative correlation between traits. Two pairs of QTLs, *qProt-1-1*/*qOil-1-2* and *qProt-6-1*/*qOil-6-1*, were associated with two-allele SNPLDB markers, Gm01_50257226 and Gm06_44035193, respectively, and significant differences were observed between both groups of accessions categorized by alleles of associated markers using *t*-tests at the level *p* ≤ 0.05 level for both seed protein and oil content. The remaining three pairs of QTLs were associated with multiple-allele SNPLDB markers, *qProt-8-1*/*qOil-8-2* with Gm08_BLOCK_16237107_16278243, *qProt-10-3*/*qOil-10-3* with Gm10_BLOCK_38679572_38679818, and *qProt-20-3*/*qOil-20-1* with Gm20_BLOCK_30995685_31177423, and there were significant differences among the accession grouped by alleles of associated markers using the least significant differences (LSDs) test at the level *p* ≤ 0.05 level for both seed protein and oil content. Of particular importance are the common QTLs *qProt-20-3*/*qOil-20-1*, which explained the largest phenotypic variation for both seed protein content (16.0%) and oil content (15.1%). Additionally, all five pairs of QTLs exhibited negative relationships between the seed protein and oil content, i.e., alleles associated with increased protein content were associated with reduced oil content and vice versa.

### 2.6. Candidate Genes Controlling Seed Protein and Oil Content

For seed protein content, 55 candidate genes in 18 of the 26 detected QTLs were inferred, with cumulative *R^2^* values accounting for 42.3% of phenotypic variation (Table S1). In the case of seed oil content, 51 candidate genes in 17 of the 23 detected QTLs were inferred, with *R^2^* values accounting for 43.6% of phenotypic variation (Table S2). Gene ontology (GO) enrichment analysis was performed using the AgriGO analysis tool (http://bioinfo.cau.edu.cn/agriGO/); 24 genes for seed protein content (Figure S3A) and 21 genes for seed protein content (Figure S3B) were annotated and classed into three GO categories, namely, biological process, cellular component, and molecular function.

## 3. Discussion

### 3.1. Efficient Multi-Locus GWAS Procedure for Dissecting the Genetic Architecture of Complex Traits

The main objective of genome-wide association studies is to identify genomic loci underlying a given trait and to dissect its genetic architecture, including the number of loci and their respective contributions to the phenotype [36]. Association mapping is a high-resolution procedure of mapping QTLs based on linkage disequilibrium and holds promise for the dissection of complex genetic traits [12]. The mixed linear model (MLM) [37], which fits the population structure (Q) or the principal components (PCs) with kinship (K), is the most popular association mapping procedure [25]. However, the MLM procedure is based on a single-locus model that tests one marker at a time, and the accumulated contribution of the detected QTLs may be inflated, leading to the overflowing heritability problem. If a stringent, experiment-wide multiple testing correction is used, false-negative results may occur with some small-effect loci being rejected [19,22]. Previous studies have proposed several multi-locus GWAS procedures to improve the power and accuracy of QTLs detection, such as the multi-locus mixed-model (MLMM) [20] and the multi-locus random-SNP effect mixed linear model (mrMLM) [19]. One evident advantage of such procedures is that multiple testing correction is not required because of the built-in experiment-wide criterion under the multi-locus model, but such procedures are only adaptable to bi-allelic SNP marker studies and fail to detect the multi-allelic variation in germplasm populations. Fortunately, the RTM-GWAS [22] procedure, based on multi-allelic SNPLDB markers, may fit the multi-allele property of complex traits in germplasm accessions well. The RTM-GWAS procedure has been widely applied to identify QTLs for soybean agronomic and seed composition traits in germplasm populations [17,18,23,24], nested association mapping populations [25,26], and recombinant inbred line populations [27].

In this study, we utilized the RTM-GWAS procedure to dissect the genetic architecture of seed component traits in soybean from the Yangtze and Huaihe River Valleys in China, and identified 26 and 23 QTLs accounting for 58.3% and 53.1% of phenotypic variation for seed protein and oil content (Table 2 and Table 3), respectively. We also performed the mrMLM procedure based on SNP markers, and 5 and 10 SNPs were identified with 23.6% and 48.4% phenotype variation contribution for seed protein (Table S3) and oil content (Table S4), respectively. Among the results of the two multi-locus GWAS procedures, only two SNPs identified by mrMLM were located close to the genomic regions of 2 QTLs identified by RTM-GWAS for seed oil content, Gm20_31299487, which explained the largest contribution to phenotypic variation (Table S4), however no common locus was identified with the two procedures for seed protein content. Although RTM-GWAS detected more QTLs and accounted for higher phenotypic variation, the comparison between the two procedures was not completely identical because multi-allelic SNPLDB markers may provide more allele information than bi-allelic SNP markers. As quantitative traits, such as seed protein and oil content, are controlled by multiple genes, and the number of markers is much higher than the sample size, it is feasible to simultaneously perform multiple GWAS procedures, particularly multi-locus procedures.

### 3.2. Previously Reported and Novel QTLs Detected with Multi-Locus GWAS Analysis

Seed protein and oil content in soybean are complex quantitative traits controlled by many genetic loci, with the majority of loci displaying minor effects [7,8]. Previous studies have identified a large number of QTLs for protein and oil content of soybean using linkage mapping in biparental populations [5,38,39] and association mapping in natural populations [4,17,18,31]. However, known QTLs should be validated before they are incorporated into marker-assisted breeding programs. Accordingly, a comparison between QTLs for the two seed component traits in this study and those in previous studies was conducted based on the physical regions of associated markers (Glyma. Wm82.a1.v1.1).

Of the 26 QTLs identified for seed protein content in our study, 19 of them were located in or around 54 QTLs found by linkage mapping in the SoyBase database and 6 QTLs were consistent with 12 QTLs reported previous in GWAS (Table 2). Of those, *qProt-20-3*, which explained the largest phenotypic contribution, corresponded to seven previously reported QTLs found by linkage mapping in biparental populations, including Seed protein 31-1 [40], cqSeed protein-003 [41], Seed protein 1-1 and 1-2 [42], Seed protein 39-4 [38], Seed protein 34-11 [39], and Seed protein 15-1 [43] and was consistent with five previously reported QTLs found by GWAS in natural populations, including Gm20_29983050 [4], Gm20_30619328 [31], Gm20_30696195−30779755 [17], Gm20_31150279 [16], and Gm20_31610452 [29] (Figure S4). The QTL *qProt-7-1*, which explained 4.7% of phenotypic variation, corresponded to Seed protein 24-4 [44], Seed protein 33-5 [5], cqSeed protein-009 [9], and Gm07_9512225 [4]. The QTL *qProt-15-1* corresponded to Seed protein 30-3 [45], Gm15_3828443 [30], Gm15_3919945 [29], and Gm_4026372 [4]. The QTL *qProt-6-2* corresponded to Seed protein 13-2 [46], Seed protein 24-1 [44], and Gm06_46040638 [16]. The QTL *qProt-4-3* corresponded to Seed protein 36-4 [11] and Gm04_ 39785974−39981932 [17]. The QTL *qProt-10-1* corresponded to Seed protein 21-5 [47] and Gm10_1397410 [4]. In addition, seven of the QTLs identified for seed protein content were novel: *qProt-8-1*, *qProt-13-1*, *qProt-17-1*, *qProt-18-1*, *qProt-19-1*, *qProt-19-2,* and *qProt-20-4*.

Among the 23 QTLs identified for seed oil content, 18 QTLs were consistent with 39 QTLs found previously by linkage mapping in the SoyBase database, seven QTLs were related to 12 QTLs found in previous GWAS (Table 3). Of these, QTL *qOil-20-1*, which accounted for the largest phenotypic contribution, corresponded to six previously reported QTLs found by linkage mapping, including Seed oil 2-1 and 2-2 [42], Seed oil 15-1 [43], Seed oil 24-30 [48], mqSeed Oil-020 [49], and cqSeed oil-004 [41], and was consistent with five previously reported QTLs found by GWAS, including Gm20_29983050 [4], Gm20_30619328 [31], Gm20_31150279 [16,29], Gm20_31164168 [35], and Gm20_ 31674614−31694438 [18]. The QTL *qOil-8-1* corresponded to Seed oil 30-3 [50], Seed oil 34-1 [45], mqSeed Oil-004 [49] and Gm08_13672776 [32]. The QTL *qOil-15-1* corresponded to Seed oil 27-2 [51], Seed oil 39-8 [52], and Gm15_11057018−11156139 [34]. The QTL *qOil-16-1* corresponded to Seed oil 5-2 [53], Seed oil 39-12 [52], Seed oil 43-19 [11], and Gm16_ 31506333−31515376 [18]. The QTL *qOil-17-1* corresponded to Seed oil 23-3 [44] and Gm17_5042611 [4]. Furthermore, this study also identified three novel QTLs seed oil content: *qOil-4-1*, *qOil-8-2* and *qOil-10-3*.

There were six QTLs for seed protein content and five QTLs for seed oil content that were previously reported both by linkage mapping and GWAS in different populations with different genetic backgrounds, and the most impactful and stable QTLs should be given priority for gene cloning and marker assistant selection in future breeding programs. As a multi-locus GWAS procedure, RTM-GWAS may be feasible and reliable to dissect the genetic architecture of complex quantitative traits.

### 3.3. Candidate Genes for Seed Protein and Oil Content for Further Study

Candidate gene analysis would be needed for further gene cloning and functional validation. From the detected QTLs, 55 and 51 candidate genes were identified for seed protein and oil content, respectively, and these genes were closely or distantly related to soybean seed protein and oil content. The genomic region (24.5 to 32.9 Mb, 8.4 Mb) on chromosome 20 is a particularly attractive major common QTL for seed protein and oil content [4,16,54]. The co-localization of QTL regions for these two seed component traits could result from pleiotropy or closely linked genes within the same region [55]. Bandillo et al. [16] refined the candidate genes for protein and oil in the 8.4 Mb region, and hypothesized three plausible candidate genes involved in these traits: *Glyma20g21030*, which is annotated as an ammonium transporter involved in embryo development, *Glyma20g21361*, which is annotated as a conserved oligomeric complex involved in the intra- and intercellular vesicle-mediated transfer and storage of proteins, and *Glyma20g21780*, which is annotated as a signal transduction histidine kinase involved in signal transduction [54]. In this study, the major common QTLs on chromosome 20 were *qProt-20-3*/*qOil-20-1*, which were identified in the same region (Gm20_ 30995685−31177423 bp). The co-localization of QTL regions for seed protein and oil content were similar to those reported by Bandillo et al. [16] and Hwang et al. [4], and *Glyma20g21693* and *Glyma20g21726* were considered the important genes within this region. *Glyma20g21693* is annotated as a subtilase family protein, which is involved in proteolysis and in the negative regulation of catalytic activity. *Glyma20g21726* is annotated as an aldehyde dehydrogenase (ALDH12A1), which is involved in the proline metabolic process and in oxidation-reduction process (SoyBase). In rice, aldehyde dehydrogenase is needed for seed maturation by detoxifying aldehydes generated by lipid peroxidation [56]. Further, pathway analysis showed that gene *Glyma04g03180* (*qProt-4-1*) is involved in the protein catabolic process and Golgi vesicular transport, *Glyma10g29020* (*qProt-10-2*) is involved in vesicle-mediated transport and amino acid import, *Glyma10g29970* (*qProt-10-3*) is involved in amino acid transport and embryo development, and *Glyma19g31120* (*qProt-19-2*) is involved in the glutamate biosynthetic process and nitrogen compound metabolic process. While *Glyma08g21340* (*qOil-8-2*) is involved in very long-chain fatty acid metabolic process and lipid metabolic process, *Glyma08g21530* (*qOil-8-2*) is involved in fatty acid biosynthetic process and embryo development (SoyBase). The eight genes mentioned above could be promising candidate genes for improving soybean seed nutrients. The results indicated that seed protein and oil content were complex traits that involve a series of biochemical pathway-related genes. Therefore, further studies are needed to validate the functions of candidate genes of these two seed component traits in soybean.

## 4. Materials and Methods

### 4.1. Plant Materials and Field Experiments

An association panel of 279 soybean accessions was selected from the Yangtze-Huai soybean breeding germplasm population, which was obtained from National Center for Soybean Improvement, Nanjing Agricultural University, Nanjing, China. The germplasm population was originally reported by Li et al. [28] and was used to identify genetic loci and candidate genes associated with resistance to *Phytophthora sojae* via association analysis. As a highly important parental source in soybean breeding programs, this germplasm population was selected for use in the present study.

The association panel was planted in a randomized complete block design with three replicates in a single row plot with a 1.0-m row length and 0.5-m row spacing. The field experiments were conducted at Huaiyin Institute of Agricultural Sciences of Xuhuai Region of Jiangsu, Huai’an, China (latitude 33°31′ N; longitude 119°01′ E) during 2015 and 2016. The field management was conducted under local cultural practice.

### 4.2. Phenotypic Evaluation and Statistical Analysis

The seed protein and oil content were quantified by near-infrared reflectance (NIR) spectroscopy DA-7200 (Perten Instruments, Huddinge, Sweden) using approximately 15−20 g whole seeds. The wavelength range covered was from 950 to 1650 nm. Prior to the this experiment, the calibration curve of NIR spectroscopy was established using about 1000 soybean samples, whose protein and oil content ranged from 33% to 48% and 16% to 25% of seed dry mass, respectively. The mean value of three scans of each sample was used in data analysis. The seed protein and oil content were reported as the relative percentage of seed weight.

For ANOVA, the General Linear Models procedure (PROC GLM) in SAS 9.4 (SAS Institute, Cary, NC, USA) was used with the genotype, environment, replication within environment and genotype × environment as random effects. The variance components were estimated using PROC VARCOMP of SAS 9.4 with the Type 1 error method. Broad-sense heritability (*h^2^*) was calculated as $h^{2}=\sigma_{g}^{2}/(\sigma_{g}^{2}+\sigma_{ge}^{2}/n+\sigma_{e}^{2}/nr)$, where $\sigma_{g}^{2}$, $\sigma_{ge}^{2}$, and $\sigma_{e}^{2}$ are genotype, genotype by environment interaction, and error variance, respectively, *n* is the number of environments, and *r* is the number of replicates.

### 4.3. SNP Genotyping and SNPLDB Marker Construction

Genotype data were obtained from Li et al. (2016). Restriction site-associated DNA sequencing (RAD-seq) was used for SNP genotyping for the association panel. The input data for SNP calling was prepared by SAMtools (version 0.1.8) [57] and then SNP calling was conducted with realSFS (version 0.983), based on the Bayesian estimation of site frequency at each site. Quality control was performed by eliminating monomorphic markers, markers with MAF < 5%, and markers with a missing rate higher than 10%. The fastPHASE software [58] was used for SNP imputation after heterozygous alleles were turned into missing alleles. A total of 59,845 SNPs with MAF ≥ 5% were used for further analysis in the present study. The linkage disequilibrium decay of the association panel was approximately 480 kb, where the linkage disequilibrium parameter (*r^2^*), which was used to estimate the degree of linkage disequilibrium between pair-wise SNPs, dropped to half its maximum value [28].

To fit the property of multiple alleles per locus in the association panel, we grouped tightly linked sequential SNPs into SNP linkage disequilibrium blocks (designated as the SNPLDBs) to form markers composed of multi-allelic haplotypes. The different combinations of linked SNPs in a block could be considered as multiple alleles. The SNPLDB markers may consist of multiple SNPs (multiple-allele) or only a single SNP (two-allele). The SNPLDB marker construction was implemented with the default parameters in RTM-GWAS software v1.2, which is publicly available at https://github.com/njau-sri/rtm-gwas. SNPLDB marker construction was performed as detailed by He et al. [22]. In total, 59,845 SNPs and 8148 SNPLDBs derived from them were identified in the association panel.

### 4.4. Genetic Diversity and Population Structure Analysis Based on SNPLDB Markers

The genetic diversity of the association panel, including the MAF, genetic richness (number of alleles) and polymorphic information content (PIC) were calculated using PowerMarker version 3.25 software [59].

The population structure of the association panel was inferred using STRUCTURE 2.3.4 software [60] with the Bayesian Markov Chain Monte Carlo (MCMC) model. The K value (number of subpopulations) was set from 1 to 10 using a burn-in of 10,000, a run length of 20,000, and each K value was obtained with three independent runs. For determination of the optimal number of subpopulations (K), the delta K value (ΔK) was estimated as described [61] employing the web-based program Structure Harvester v0.6.94 (available at http://taylor0.biology.ucla.edu/structureHarvester/) [62]. The subpopulation structure was visualized by bar plot using the web application STRUCTURE PLOT v2.0 (available at http://btismysore.in/strplot) [63].

Based on multi-allelic SNPLDB markers, a GSC matrix was constructed to estimate the comprehensive population structure as described in detail previously [22]. The GSC between two individuals is defined as the proportion of loci that are in identity-by-state. The scatter plot of the top three eigenvectors of the GSC matrix was produced with the R package scatterplot3d [64]. The GSC matrix based on genome-wide SNPLDBs was visualized in a heatmap along with the hierarchical cluster dendrogram constructed using the average (UPGMA) method and the R function heatmap.2 in the gplots package (http://cran.r-project.org/package=gplots).

### 4.5. Multi-Locus Genome-Wide Association Study

The RTM-GWAS procedure [22] was performed to dissect the genetic architecture underlying seed protein and oil content, based on 8148 SNPLDB markers and the plot-based whole protein and oil content data set under two environments (i.e., all the plot values). The association study was conducted in two stages. In the first stage, a single-locus association test based on the simple linear model was employed to preselect markers, and in the second stage, stepwise regression under the multi-locus multi-allele model featured with forward selection and backward elimination was applied to the preselected markers to detect genome-wide QTLs. At both stages, the 10 eigenvectors with the largest eigenvalues of the GSC matrix calculated from the genome-wide SNPLDBs were incorporated as covariates for population structure correction. A significance level of *p* ≤ 0.05 was used for the preselection of markers and a level of *p* ≤ 0.01 was used for the stepwise regression. The conservative Bonferroni criterion was not necessary to apply here because the built-in experiment-wide threshold was incorporated into the multi-locus model. The genetic contribution (*R^2^*), and allele effects per QTL were obtained from the stepwise regression in the second stage of the association analysis, and the total genetic contribution (*R^2^*) of all QTLs of one trait is the sum of *R^2^* of all QTLs. The detected QTLs were named as *qPro* and *qOil* for seed protein and oil content, followed by chromosome number and a serial order on the same chromosome [65].

### 4.6. Candidate Gene Prediction

The candidate genes from the detected QTLs were inferred according to the SoyBase database (http://soybase.org). Firstly, the annotated genes were searched within the interval of associated SNPLDBs or its flanking SNPLDBs when there was no gene inside the SNPLDB. Secondly, to identify the candidate genes from annotated genes, the Chi-square test was conducted for the association between the alleles of the detected SNPLDBs and SNPs in the annotated genes at a significance level of *p* ≤ 0.0001. An annotated gene was considered the candidate gene if all SNPs in the gene were significantly associated with those in the detected SNPLDB. If multiple genes met the criteria, the gene with the molecular function most closely related to seed protein and/or oil content was selected according to the gene ontology descriptions in SoyBase. The Glyma. Wm82.a1.v1.1 gene model from SoyBase was used and retrieved for gene calls and annotations.

## 5. Conclusions

Seed protein and oil content are two valuable quantitative traits that are controlled by multiple genes in soybean. In this study, the multi-locus RTM-GWAS procedure was utilized to dissect the genetic architecture of seed protein and oil content in the soybean association panel from the Yangtze and Huaihe River Valleys in China. We successfully identified many previously reported QTLs associated with seed protein and oil content as well as a few novel QTLs, and we obtained the co-location of QTLs. These results will facilitate marker-assisted selective breeding and positional cloning of the causal genes for soybean protein and oil content traits.

## Figures and Tables

**Table 1:** Descriptive statistics, broad sense heritability and *F*-value from ANOVA for seed protein and oil content in the soybean association panel.

Trait	Year	Mean ± SD (%)	Range (%)	Heritability (%)	*F*-Values from ANOVA		
					Accession	Environment	Accession × Environment
Protein content	2015	42.6 ± 2.1	37.2–47.6	80.4	5.10 ***	0.08 ^ns^	1.50 ***
	2016	42.6 ± 2.5	36.1–49.3				
Oil content	2015	18.5 ± 1.2	15.2–21.4	79.0	4.77 ***	1.91 ^ns^	1.39 **
	2016	18.2 ± 1.3	14.8–21.1				

ns, not significant; *** and **, significant at *p* < 0.001 and *p* < 0.01, respectively.

**Table 2:** Quantitative trait loci (QTLs) associated with the seed protein content detected in the soybean association panel.

QTL	SNPLDB ^a^	Allele Number	Log_10_ *P*	*R^2^* (%)	QTL in SoyBase ^b^	QTL in Previous GWAS ^c^
*qProt-1-1*	Gm01_50257226	2	11.7	2.8	Seed protein 13-1,31-3, 36-9,36-10	
*qProt-2-1*	Gm02_BLOCK_34241156_34302885	3	3.8	1.0	Seed protein 27-1	
*qProt-3-1*	Gm03_46437265	2	2.9	0.6	Seed protein 27-4	
*qProt-4-1*	Gm04_BLOCK_2157397_2345500	5	8.2	2.4	Seed protein 4-4,9-2, 36-5,36-6	
*qProt-4-2*	Gm04_8725710	2	2.1	0.4	Seed protein 7-2,19-1	
*qProt-4-3*	Gm04_BLOCK_39987192_40167695	7	2.8	1.2	Seed protein 36-4	Zhang et al. [17]
*qProt-6-1*	Gm06_44035193	2	5.2	1.1	Seed protein 36-7,36-8, cqSeed protein-012	
*qProt-6-2*	Gm06_BLOCK_46724398_46724570	3	5.3	1.4	Seed protein 13-2,24-1	Bandillo et al. [16]
*qProt-7-1*	Gm07_BLOCK_8657174_8845556	7	15.2	4.7	Seed protein 24-4,33-5, cqSeed protein-009	Hwang et al. [4]
*qProt-8-1*	Gm08_BLOCK_16237107_16278243	11	24.1	8.0		Novel
*qProt-8-2*	Gm08_BLOCK_42026048_42200505	6	3.2	1.2	Seed protein 3-1,21-1	
*qProt-9-1*	Gm09_BLOCK_31004337_31004679	2	3.4	0.7	Seed protein 36-28,36-29, 36-30,37-10,47-2	
*qProt-10-1*	Gm10_BLOCK_856393_1044958	5	3.0	1.0	Seed protein 21-5	Hwang et al. [4]
*qProt-10-2*	Gm10_BLOCK_37907908_38079904	4	13.2	3.6	Seed protein 27-5,36-39, 36-40,40-1	
*qProt-10-3*	Gm10_BLOCK_38679572_38679818	4	5.1	1.5	Seed protein 12-5,36-38	
*qProt-13-1*	Gm13_BLOCK_2883529_3082036	4	5.3	1.5		Novel
*qProt-15-1*	Gm15_3633885	2	3.0	0.6	Seed protein 30-3	Hwang et al. [4]; Vaughn et al. [29]; Zhang et al. [30]
*qProt-15-2*	Gm15_31011761	2	5.9	1.3	Seed protein 27-2	
*qProt-17-1*	Gm17_BLOCK_6574500_6577175	3	7.8	2.0		Novel
*qProt-18-1*	Gm18_BLOCK_35961794_36027902	3	3.0	0.8		Novel
*qProt-19-1*	Gm19_37500961	2	3.0	0.6		Novel
*qProt-19-2*	Gm19_BLOCK_38886258_38944167	4	3.1	0.9		Novel
*qProt-20-1*	Gm20_5531497	2	2.5	0.5	Seed protein 1-3,1-4, 3-12,10-1,11-1,30-1, 36-26,37-8,47-8	
*qProt-20-2*	Gm20_BLOCK_27111387_27111623	3	3.6	0.9	Seed protein 1-1,1-2, 15-1,26-5,31-1,34-11, 39-4,cqSeed protein-003	
*qProt-20-3*	Gm20_BLOCK_30995685_31177423	4	56.1	16.0	Seed protein 1-1,1-2, 15-1, 31-1,34-11,39-4, cqSeed protein-003	Hwang et al. [4]; Vaughn et al. [29]; Bandillo et al. [16]; Sonah et al. [31]; Zhang et al. [17]
*qProt-20-4*	Gm20_BLOCK_43288485_43465351	4	5.7	1.6		Novel
**Total**	**26**	**98**		**58.3**	**19 (54)**	**6 (12)**

^a^ SNPLDB: SNP linkage disequilibrium block; for example, it is designated as Gm01_50257226 if it contains only one SNP where Gm01 denotes chromosome 1 and 50257226 is its physical position in bp; it is designated as Gm02_BLOCK_34241156_34302885 if it contains multiple SNPs where Gm02 denotes chromosome 2 and 34241156_34302885 is its physical region in bp. ^b^ QTL in SoyBase: 19 (54) indicates that 19 QTLs detected in this study were located in or around 54 QTLs by linkage mapping in SoyBase. ^c^ 6 (12) indicates that 6 QTLs detected in this study were consistent with 12 QTLs reported in previous GWAS literature and “Novel” indicates a novel QTL identified in this study.

**Table 3:** QTLs associated with the seed oil content detected in the soybean association panel.

QTL	SNPLDB ^a^	Allele No.	−Log_10_ *P*	*R^2^* (%)	QTL in SoyBase ^b^	QTL in Previous GWAS ^c^
*qOil-1-1*	Gm01_BLOCK_41522087_41713586	6	5.9	1.9	Seed oil 24-19,39-5, mqSeed Oil-009	
*qOil-1-2*	Gm01_50257226	2	9.5	2.1	Seed oil 42-21	
*qOil-3-1*	Gm03_BLOCK_4553710_4553720	3	5.0	1.2	Seed oil 24-5,39-14	
*qOil-3-2*	Gm03_BLOCK_11906061_11923443	3	7.1	1.7	Seed oil 39-15, cqSeed oil-005	
*qOil-4-1*	Gm04_21748147	2	5.3	1.1		Novel
*qOil-4-2*	Gm04_41026444	2	3.2	0.6		Zhang et al. [18]
*qOil-4-3*	Gm04_BLOCK_47957394_47957714	2	2.3	0.4	mqSeed Oil-007	
*qOil-6-1*	Gm06_44035193	2	3.3	0.6	Seed oil 23-1,31-2, 33-1,38-2	
*qOil-7-1*	Gm07_38954920	2	3.0	0.6	Seed oil 34-7	
*qOil-8-1*	Gm08_BLOCK_14242705_14306849	6	10.7	3.2	Seed oil 30-3,34-1, mqSeed Oil-004	Han et al. [32]
*qOil-8-2*	Gm08_BLOCK_16237107_16278243	11	15.4	5.2		Novel
*qOil-8-3*	Gm08_BLOCK_18015046_18031943	4	5.7	1.6		Zhang et al. [33]
*qOil-10-1*	Gm10_BLOCK_5509737_5559675	3	4.1	1.0	Seed oil 34-6,43-33, 43-34	
*qOil-10-2*	Gm10_22446436	2	2.9	0.6	Seed oil 19-3	
*qOil-10-3*	Gm10_BLOCK_38679572_38679818	4	9.9	2.6		Novel
*qOil-10-4*	Gm10_BLOCK_46662161_46730774	3	18.4	4.6	Seed oil 29-3	
*qOil-13-1*	Gm13_BLOCK_30751302_30790418	6	3.8	1.3	Seed oil 37-8,38-4	
*qOil-15-1*	Gm15_BLOCK_10984687_11112792	3	2.6	0.6	Seed oil 27-2,39-8	Zhou et al. [34]
*qOil-16-1*	Gm16_31945745	2	11.7	2.7	Seed oil 39-12	Zhang et al. [18]
*qOil-17-1*	Gm17_BLOCK_5346273_5356960	2	8.0	1.8	Seed oil 23-3	Hwang et al. [4]
*qOil-17-2*	Gm17_9078832	2	5.1	1.1	Seed oil 43-12	
*qOil-18-1*	Gm18_BLOCK_11985000_12125810	4	4.9	1.4	Seed oil 27-10,43-16	
*qOil-20-1*	Gm20_BLOCK_30995685_31177423	4	55.0	15.1	Seed oil 2-1,2-2,15-1, 24-30,mqSeed Oil-020, cqSeed oil-004	Hwang et al. [4]; Vaughn et al. [29]; Bandillo et al. [16]; Sonah et al. [31]; Cao et al. [35]; Zhang et al. [18]
**Total**	**23**	**80**		**53.1**	**18 (39)**	7 **(12)**

^a^ SNPLDB: SNP linkage disequilibrium block; for example, it is designated as Gm01_BLOCK_41522087_41713586 if it contains multiple SNPs where Gm01 denotes chromosome 1 and 41522087_41713586 is its physical region in bp; and it is designated as Gm01_50257226 if it contains only one SNP where Gm01 denotes chromosome 1 and 50257226 is its physical position in bp. ^b^ 18 (36) indicates that 18 QTLs detected in this study are located in or around 36 QTLs by linkage mapping in SoyBase. ^c^ 7 (12) indicates that 7 QTLs detected in this study were consistent with 12 QTLs QTLs reported in previous GWAS literature and “Novel” indicates a novel QTL identified in this study.

**Table 4:** *T*-test or multiple comparison among means of accessions grouped by the allele of QTLs associated with both seed protein and oil content.

QTL	SNPLDB	Allele	Frequency	Protein Content (%) ^a^	Oil Content (%) ^a^	Protein vs. Oil Relationship ^b^
*qProt-1-1/qOil-1-2*	Gm01_50257226	A	197	43.0 *	18.2 *	Negative
		C	82	41.8	18.8	
*qProt-6-1/qOil-6-1*	Gm06_44035193	C	154	43.2 *	18.1 *	Negative
		T	125	41.9	18.7	
*qProt-8-1/qOil-8-2*	Gm08_BLOCK_16237107_16278243	CGCCATT	3	45.6 a	16.8 f	Negative
		CGCTGCA	6	45.3 a	17.1 ef	
		TGTCGTT	4	44.6 ab	17.2 ef	
		CGCTATT	4	43.8 bc	18.0 cde	
		CGCTACA	82	43.7 bc	17.8 de	
		CGTCGTT	19	43.1 bcd	18.2 cd	
		CGCCGCA	5	42.4 cde	18.3 bcd	
		TGCCGTT	76	42.0 de	18.6 abcd	
		CATCGTT	57	41.8 de	18.8 abc	
		CGCTGTT	5	41.0 e	19.1 ab	
		CGCCGTT	18	40.9 e	19.3 a	
*qProt-10-3/qOil-10-3*	Gm10_BLOCK_38679572_38679818	GCCC	5	46.4 a	16.1 b	Negative
		ACTC	212	42.8 b	18.3 a	
		GCCT	44	42.2 bc	18.6 a	
		GTCC	18	41.2 c	18.9 a	
*qProt-20-3/qOil-20-1*	Gm20_BLOCK_30995685_31177423	CACCCAGGAATCACGGACGCGC	20	45.0 a	17.1 c	Negative
		TTATTCAATGCTGTATCTATAT	70	44.0 b	17.7 b	
		CTATTCAATGCTGTATCTATAT	109	42.1 c	18.6 a	
		CTATTCAATGCTGTATCTATGT	80	41.6 c	18.9 a	

^a^*t*-tests were utilized to test the differences between the accessions grouped by alleles of associated SNPLDB markers comprising two alleles; such markers include Gm01_50257226 and Gm06_44035193. * indicates that means of seed protein and oil content is significantly different at the level *p* ≤ 0.05. Least significant differences (LSDs) were utilized to test the differences among the accessions grouped by alleles of associated SNPLDB markers comprising multiple alleles; such markers include Gm08_BLOCK_16237107_16278243, Gm10_BLOCK_38679572_38679818, and Gm20_BLOCK_30995685_31177423. Means of seed protein and oil content with the same letter are not significantly different at the level *p* ≤ 0.05 (in lowercase). ^b^ Relationship between the seed protein and oil content based on the alleles of associated SNPLDB markers. A negative relationship means that the allele associated with increased protein content was associated with reduced oil content and vice versa.

## Supplementary Materials

Supplementary materials can be found at https://www.mdpi.com/1422-0067/20/12/3041/s1.

## Abbreviations

ANOVA	analysis of variance
GSC	genetic similarity coefficient
GWAS	genome-wide association study
MAF	minor allele frequency
NIR	near-infrared reflectance
PIC	polymorphic information content
QTL	quantitative trait loci
RAD-seq	restriction site-associated DNA sequencing
RIL	recombinant inbred line
RTM-GWAS	restricted two-stage multi-locus multi-allele (RTM) genome-wide association study (GWAS)
SNP	single nucleotide polymorphism
SNPLDB	SNP linkage disequilibrium block
