# Supplementary material for: Dissecting the Genetic Architecture of Seed Protein and Oil Content in Soybean from the Yangtze and Huaihe River Valleys Using Multi-Locus Genome-Wide Association Studies
Source: Int J Mol Sci. 2019 Jun 21;20(12):3041. doi: 10.3390/ijms20123041 (PMC6628128; doi:10.3390/ijms20123041)
Supplement: Supplementary file 1 [file ijms-20-03041-s001.pdf]

Supplementary material

Dissecting the Genetic Architecture of Seed Protein and Oil Content in Soybean from the Yangtze and Huaihe River Valleys Using Multi-Locus Genome-Wide Association Studies

Shuguang Li [1], Haifeng Xu [1], Jiayin Yang [1,*], Tuanjie Zhao [2,*]

[1] Huaiyin Institute of Agricultural Sciences of Xuhuai Region in Jiangsu / Huai'an Key Laboratory for Agricultural Biotechnology, Huai'an 223001, China; dawn0524@126.com (S.L.); hanksxhf@163.com (H.X.)

[2] Soybean research institution, National Center for Soybean Improvement, Key Laboratory of Biology and Genetics and Breeding for Soybean, Ministry of Agriculture, State Key Laboratory of Crop Genetics and Germplasm Enhancement, Nanjing Agricultural University, Nanjing 210095, China

* Correspondence: hynksyjy@163.com (J.Y.); tjzhao@njau.edu.cn (T.Z.)

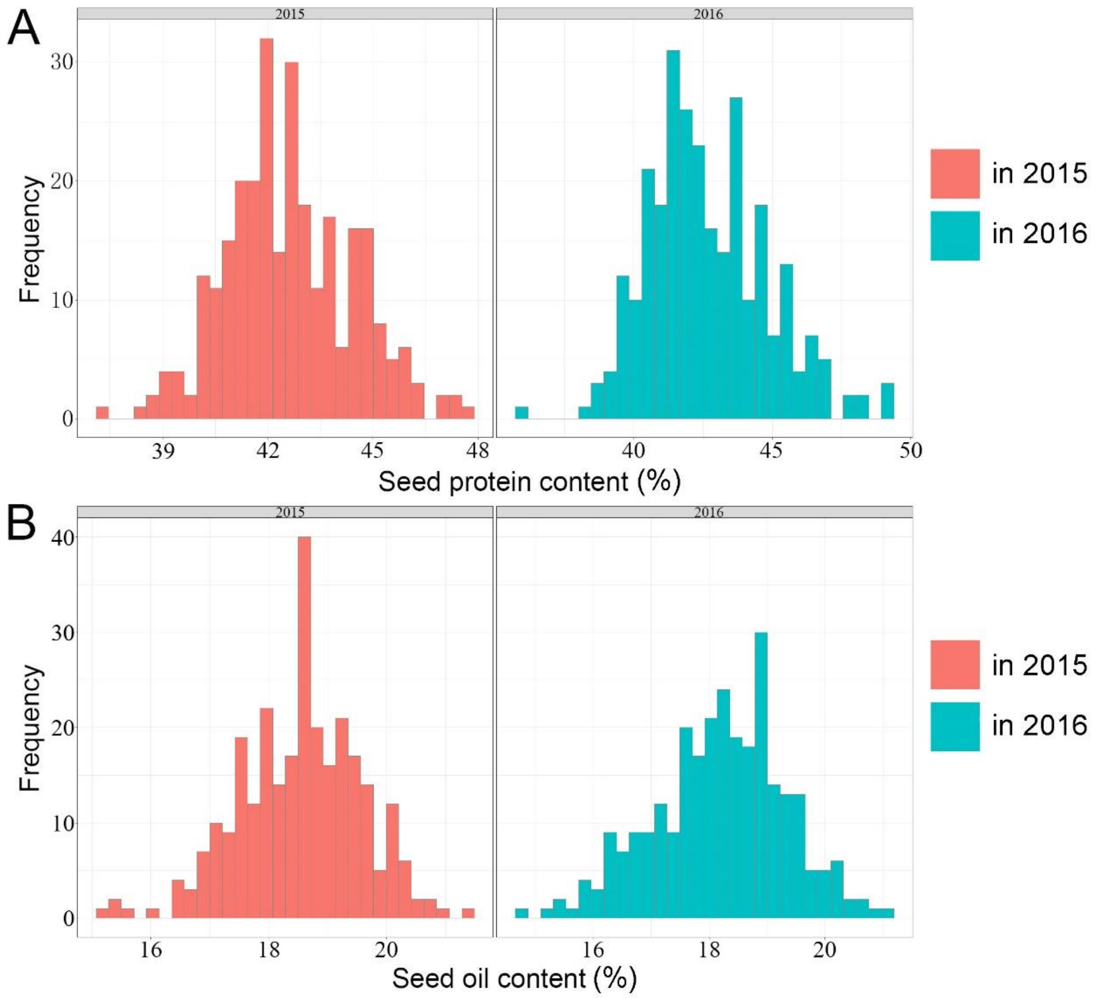

Supplementary Figure 1 Phenotypic distribution of seed protein (A) and oil (B) content in the soybean association panel

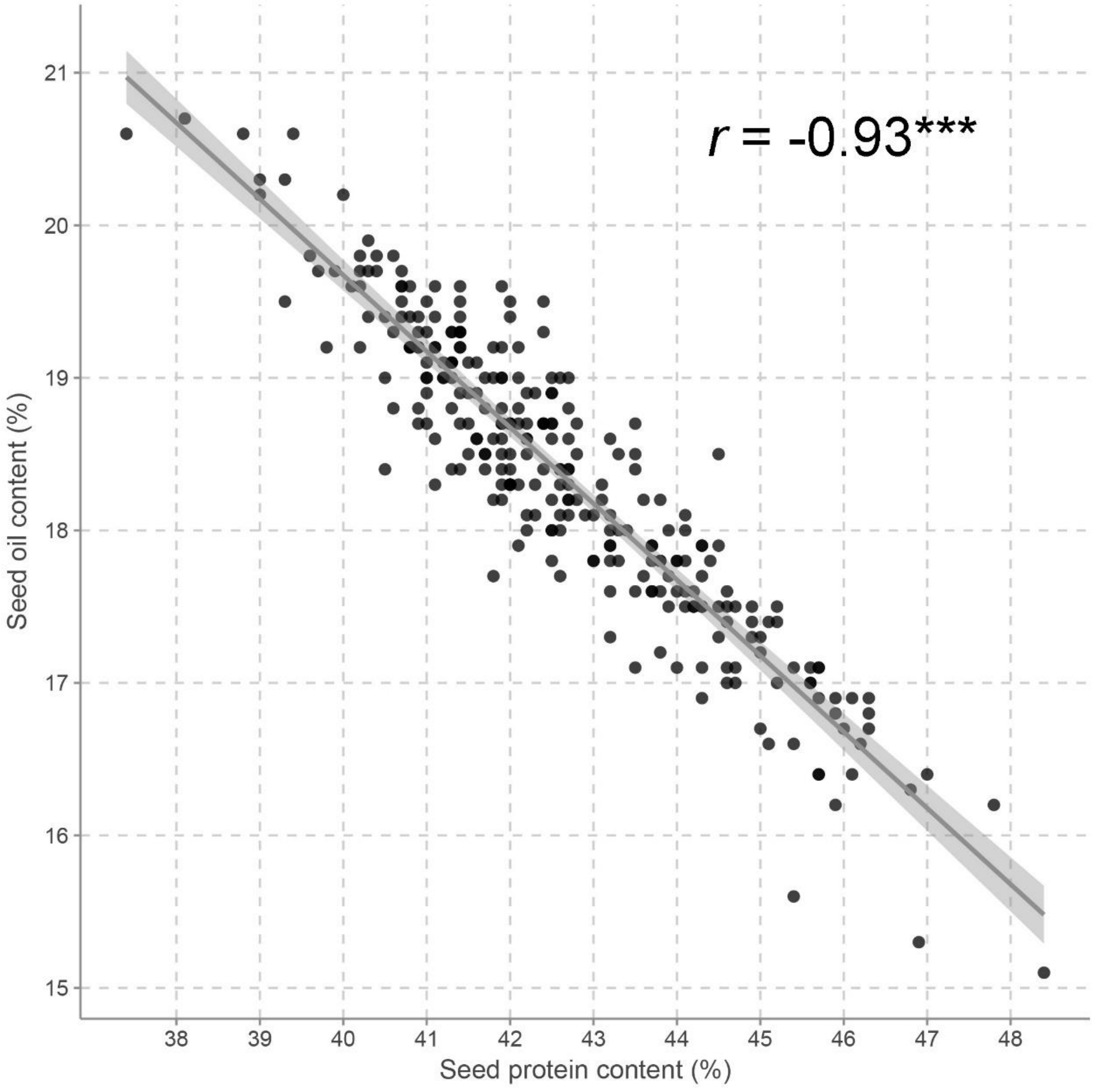

Supplementary Figure 2 Scatter plot for seed protein and oil content in the soybean association panel

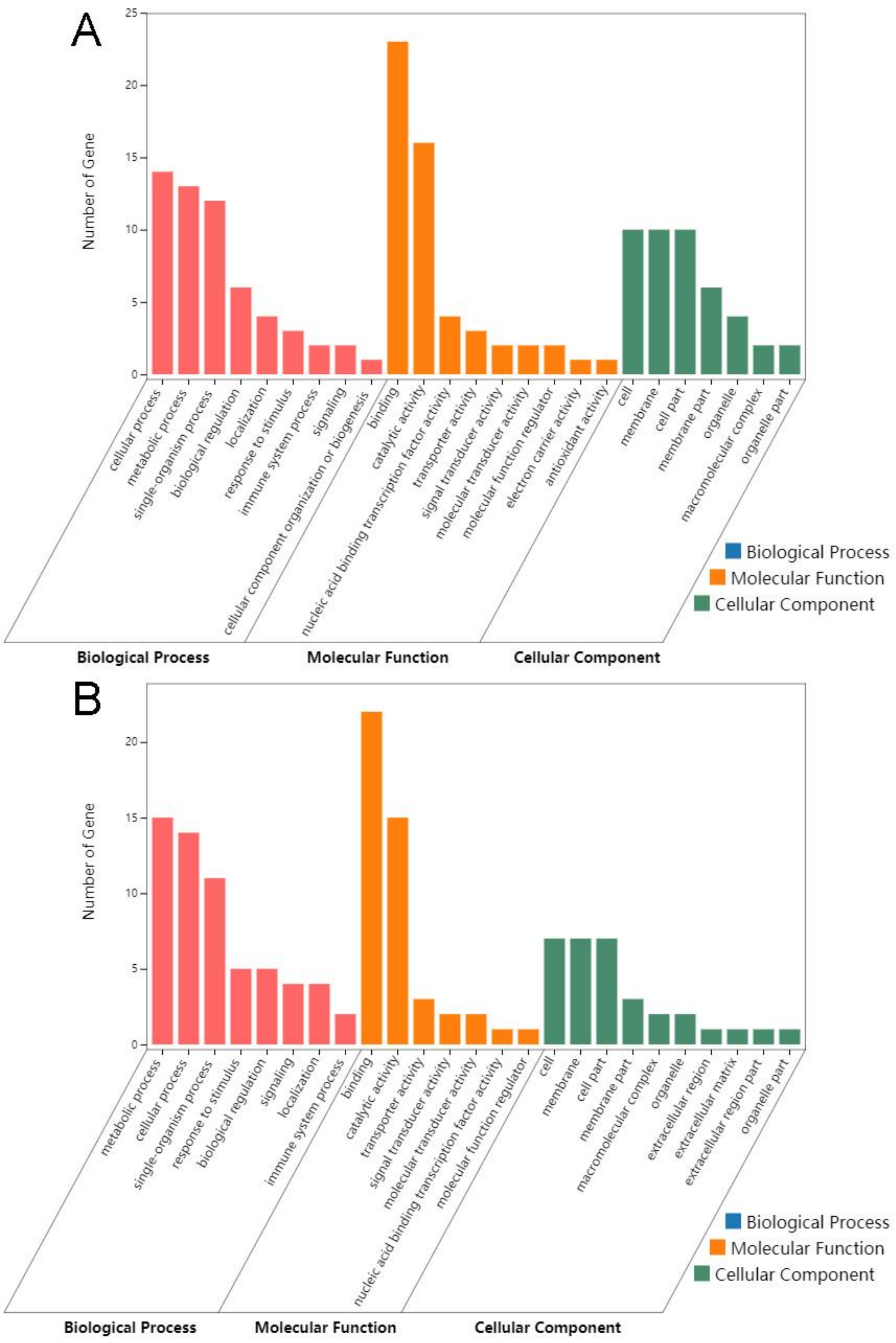

Supplementary Figure 3 Gene ontology classification of annotated genes for seed protein (A) and oil (B) content in the soybean association panel.

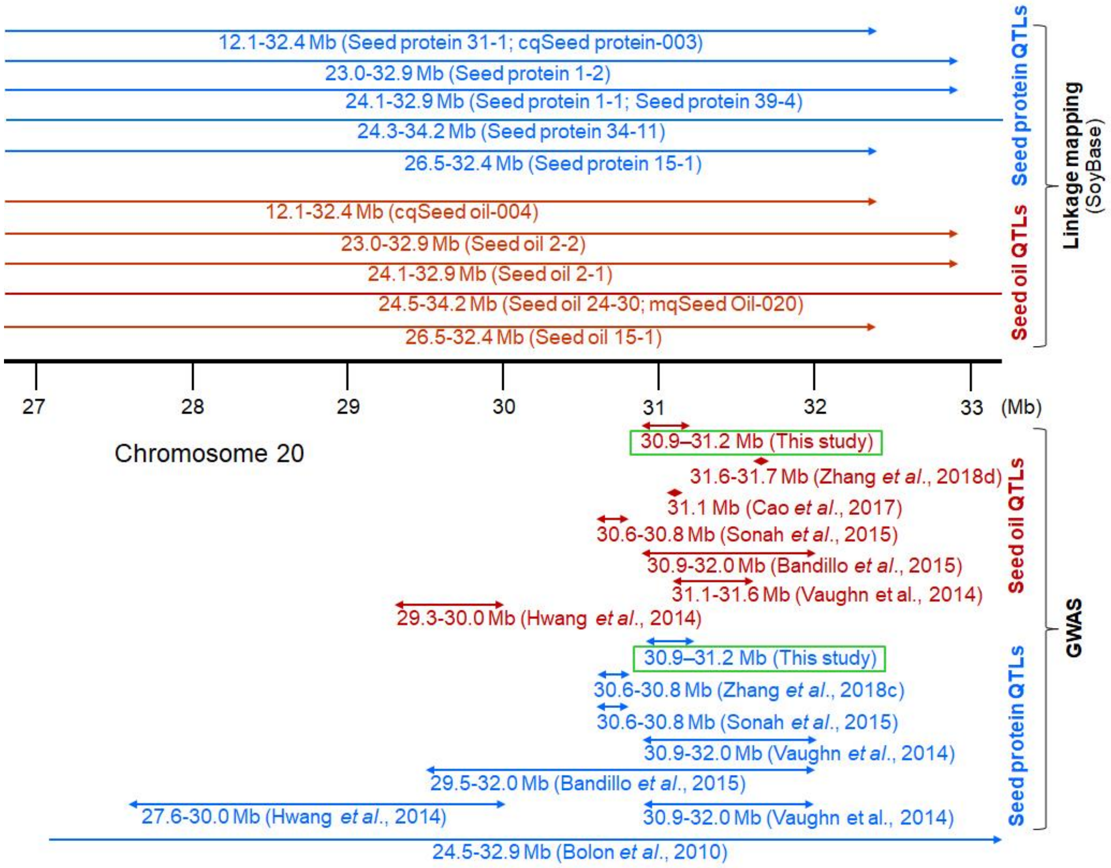

Supplementary Figure 4 The candidate region of the major pleiotropic seed protein and oil content QTLs on Chromosome 20. Previously identified QTLs of seed protein and oil content through linkage mapping were obtained from SoyBase (http://soyba se.org) and are indicated above the horizontal axis. Previously identified QTLs of seed protein and oil content through GWAS are indicated below the horizontal axis. The seed protein and oil content QTLs are shown in blue and red, respectively. This study identified a major pleiotropic seed protein and oil content QTL in the 30.9-31.2 Mb region on chromosom 20 identified by RTM-GWAS procedure.

Supplementary Table 1 The candidate genes annotated from the QTLs associated with the seed protein content detected in the soybean association panel

QTL	R^2 (%)	Gene	Chromosome	Start	End	Functional annotation [a]
qProt-2-1	1.0	*Glyma02g31440*	Gm02	34348334	34355479	NAD(P)-linked oxidoreductase superfamily protein
qProt-3-1	0.6	*Glyma03g40780*	Gm03	46431667	46441697	Ankyrin repeat family protein
qProt-4-1	2.4	*Glyma04g02850*	Gm04	2059088	2063081	Basic-leucine zipper (bZIP) transcription factor family protein
		Glyma04g02880	Gm04	2082594	2084666	Glyoxal oxidase-related protein
		Glyma04g02910	Gm04	2103499	2103663	–
		Glyma04g02980	Gm04	2155604	2159914	K-box region and MADS-box transcription factor family protein
		Glyma04g03180	Gm04	2320692	2324583	AAA-type ATPase family protein
		Glyma04g03230	Gm04	2366242	2370703	Sec14p-like phosphatidylinositol transfer family protein
qProt-4-2	0.4	*Glyma04g10451*	Gm04	8670881	8701308	CRT (chloroquine-resistance transporter)-like transporter 2
		Glyma04g10481	Gm04	8701971	8719497	Ubiquitin-protein ligase 1
		Glyma04g10510	Gm04	8725073	8727209	Target SNARE coiled-coil domain protein
qProt-6-1	1.1	*Glyma06g40780*	Gm06	43967782	43972339	Disease resistance protein (TIR-NBS-LRR class) family
		Glyma06g40820	Gm06	44001679	44004225	Disease resistance protein (TIR-NBS class)
qProt-7-1	4.7	*Glyma07g10621*	Gm07	8869326	8879181	Protein kinase superfamily protein
qProt-8-1	8.0	*Glyma08g21340*	Gm08	16195831	16198280	GDSL-like Lipase/Acylhydrolase superfamily protein
		Glyma08g21450	Gm08	16289768	16295899	Hexokinase 2
		Glyma08g21530	Gm08	16348536	16354299	NADP-malic enzyme 4
qProt-8-2	1.2	*Glyma08g42100*	Gm08	41999064	42001005	RNI-like superfamily protein
		Glyma08g42110	Gm08	42002502	42039978	Glucan synthase-like 7
		Glyma08g42170	Gm08	42127192	42132559	Protein kinase superfamily protein
		Glyma08g42230	Gm08	42188239	42197436	MEI2-like protein 5
		Glyma08g42240	Gm08	42224157	42231045	MAP kinase 20
qProt-9-1	0.7	*Glyma09g24910*	Gm09	30904477	30909668	Hydroxyproline-rich glycoprotein family protein
		Glyma09g24956	Gm09	30999671	31007110	Divalent ion symporter
qProt-10-1	1.0	*Glyma10g01180*	Gm10	878255	880592	Plant invertase/pectin methylesterase inhibitor superfamily
		Glyma10g01241	Gm10	906726	913111	Unknown protein
		Glyma10g01440	Gm10	1070032	1070748	–
		Glyma10g01450	Gm10	1069805	1073166	WRKY DNA-binding protein 23
qProt-10-2	3.6	*Glyma10g29000*	Gm10	37895786	37900936	Importin alpha isoform 2
		Glyma10g29020	Gm10	37907198	37912351	Syntaxin of plants 43
		Glyma10g29180	Gm10	38076511	38080168	Unknown function
		Glyma10g29190	Gm10	38081913	38084017	Dormancy/auxin associated family protein
		Glyma10g29280	Gm10	38162014	38169053	Respiratory burst oxidase homolog B
qProt-10-3	1.5	*Glyma10g29831*	Gm10	38598943	38610302	Mitochondrial inner membrane translocase complex, subunit Tim44-related protein
		Glyma10g29851	Gm10	38616053	38627075	ROP binding protein kinases 2

QTL	R^2 (%)	Gene	Chromosome	Start	End	Functional annotation [a]
		Glyma10g29861	Gm10	38619549	38620910	Protein of unknown function (DUF3049)
		Glyma10g29900	Gm10	38652112	38654153	Prefoldin chaperone subunit family protein
		Glyma10g29970	Gm10	38676542	38684098	Dehydroquinate dehydratase, putative / shikimate dehydrogenase, putative
		Glyma10g30040	Gm10	38744503	38746299	–
		Glyma10g30070	Gm10	38768258	38776250	Protein kinase superfamily protein
qProt-15-2	1.3	*Glyma15g28100*	Gm15	30878306	30937620	Guanyl-nucleotide exchange factors; GTPase binding; GTP binding
qProt-17-1	2.0	*Glyma17g08880*	Gm17	6573195	6574595	Nonsense-mediated mRNA decay NMD3 family protein
		Glyma17g08890	Gm17	6576987	6586049	AGAMOUS-like 8
qProt-19-1	0.6	*Glyma19g29730*	Gm19	37457990	37461737	Exostosin family protein
		Glyma19g29760	Gm19	37499170	37501892	F-box family protein
qProt-19-2	0.9	*Glyma19g31120*	Gm19	38858770	38898403	Glutamate synthase 1
qProt-20-3	16.0	*Glyma20g21693*	Gm20	31187632	31192534	Subtilase family protein
		Glyma20g21726	Gm20	31247519	31249743	Aldehyde dehydrogenase 12A1
qProt-20-4	1.6	*Glyma20g34880*	Gm20	43205506	43209164	Differentiation and greening-like 1
		Glyma20g34910	Gm20	43226352	43228993	Embryo sac development arrest 6
		Glyma20g34975	Gm20	43294976	43298657	ROP-interactive CRIB motif-containing protein 1
		Glyma20g35020	Gm20	43315854	43330441	COP1-interacting protein-related
		Glyma20g35030	Gm20	43321080	43321497	–
		Glyma20g35090	Gm20	43366905	43377339	Calcium-binding EF-hand family protein
		Glyma20g35170	Gm20	43452291	43452785	Unknown protein
**Total (18)**	**48.7**	**55**				

[a] Candidate gene annotation was obtained from SoyBase (http://www.soybase.org)

Note: Physical positions and genes are based on soybean reference genome Glyma. Wm82.a1.v1.1

Supplementary Table 2 The candidate genes annotated from the QTLs associated with the seed oil content detected in the soybean association panel

QTL	R^2 (%)	Gene	Chromosome	Start	End	Functional annotation [a]
qOil-1-1	1.9	Glyma01g30670	Gm01	41495109	41496829	PEBP (phosphatidylethanolamine-binding protein) family protein
		Glyma01g30910	Gm01	41681660	41690953	BCL-2-associated athanogene 4
		Glyma01g30982	Gm01	41712880	41714252	–
qOil-3-1	1.2	Glyma03g04340	Gm03	4465554	4469715	Protein kinase superfamily protein
		Glyma03g04420	Gm03	4551604	4558159	MATE efflux family protein
		Glyma03g04470	Gm03	4623994	4625733	Ovate family protein 1
qOil-3-2	1.7	Glyma03g09870	Gm03	11876472	11888309	protein kinase 1B
		Glyma03g10002	Gm03	11918753	11926518	Peroxisomal membrane 22 kDa (Mpv17/PMP22) family protein
qOil-4-2	0.6	Glyma04g34745	Gm04	40970963	40973184	FAR1-related sequence 5
		Glyma04g34790	Gm04	41008255	41014132	ARM repeat superfamily protein
qOil-4-3	0.4	Glyma04g42167	Gm04	47951206	47959456	ACT domain repeat 3
		Glyma04g42174	Gm04	47969886	47975364	Protein kinase protein with adenine nucleotide alpha hydrolases-like domain
		Glyma04g42240	Gm04	48012311	48014973	–
		Glyma04g42246	Gm04	48019840	48027339	Target of Myb protein 1
qOil-6-1	0.6	Glyma06g40780	Gm06	43967782	43972339	Disease resistance protein (TIR-NBS-LRR class) family
		Glyma06g40820	Gm06	44001679	44004225	Disease resistance protein (TIR-NBS class)
qOil-8-2	5.2	Glyma08g21340	Gm08	16195831	16198280	GDSL-like Lipase/Acylhydrolase superfamily protein
		Glyma08g21450	Gm08	16289768	16295899	Hexokinase 2
		Glyma08g21530	Gm08	16348536	16354299	NADP-malic enzyme 4
qOil-8-3	1.6	Glyma08g23494	Gm08	17922006	17924880	Unknown protein
		Glyma08g23640	Gm08	18014906	18017420	Unknown protein
		Glyma08g23660	Gm08	18031482	18035658	Galactose oxidase/kelch repeat superfamily protein
		Glyma08g23760	Gm08	18103043	18123371	Autoinhibited Ca(2+)-ATPase 9
qOil-10-1	1.0	Glyma10g06660	Gm10	5413833	5414572	–
		Glyma10g06670	Gm10	5414057	5421473	Protein phosphatase 2A regulatory B subunit family protein
		Glyma10g06800	Gm10	5502093	5516322	Phosphatidyl inositol monophosphate 5 kinase
		Glyma10g06820	Gm10	5553816	5569768	Glutamate-ammonia ligases;catalytics;glutamate-ammonia ligases
qOil-10-3	2.6	Glyma10g29831	Gm10	38598943	38610302	Mitochondrial inner membrane translocase complex, subunit Tim44-related protein
		Glyma10g29851	Gm10	38616053	38627075	ROP binding protein kinases 2
		Glyma10g29861	Gm10	38619549	38620910	Protein of unknown function (DUF3049)
		Glyma10g29900	Gm10	38652112	38654153	Prefoldin chaperone subunit family protein
		Glyma10g29970	Gm10	38676542	38684098	Dehydroquinate dehydratase, putative / shikimate dehydrogenase, putative
		Glyma10g30040	Gm10	38744503	38746299	–

QTL	R^2 (%)	Gene	Chromosome	Start	End	Functional annotation [a]
		Glyma10g30070	Gm10	38768258	38776250	Protein kinase superfamily protein
qOil-10-4	4.6	Glyma10g38820	Gm10	46597891	46602238	Unknown protein
		Glyma10g38890	Gm10	46638029	46641386	NAD(P)-linked oxidoreductase superfamily protein
		Glyma10g38930	Gm10	46662019	46664981	Heat shock transcription factor A6B
		Glyma10g38970	Gm10	46684505	46690367	TIFY domain/Divergent CCT motif family protein
qOil-13-1	1.3	Glyma13g27480	Gm13	30660060	30670615	Starch synthase 3
		Glyma13g27570	Gm13	30770943	30775413	RNA-binding (RRM/RBD/RNP motifs) family protein
		Glyma13g27610	Gm13	30790326	30791295	Pentatricopeptide repeat 336
qOil-15-1	0.6	Glyma15g14700	Gm15	11189089	11197781	Chaperone DnaJ-domain superfamily protein
qOil-16-1	2.7	Glyma16g27980	Gm16	31945624	31952671	WD-40 repeat family protein / notchless protein, putative
		Glyma16g28080	Gm16	31997661	32006814	Cellulose synthase 6
qOil-17-1	1.8	Glyma17g07341	Gm17	5355165	5357969	UDP-glucosyl transferase 78D2
qOil-17-2	1.1	Glyma17g11950	Gm17	8995174	9001876	Tetratricopeptide repeat (TPR)-like superfamily protein
		Glyma17g11961	Gm17	9006506	9013109	–
		Glyma17g12040	Gm17	9078459	9079741	Calcium-binding EF-hand family protein
		Glyma17g12120	Gm17	9160275	9165307	Zinc finger (ubiquitin-hydrolase) domain-containing protein
qOil-20-1	15.1	Glyma20g21693	Gm20	31187632	31192534	Subtilase family protein
		Glyma20g21726	Gm20	31247519	31249743	Aldehyde dehydrogenase 12A1
**Total (17)**	**44.2**	**51**				

[a] Candidate gene annotation was obtained from SoyBase (http://www.soybase.org)

Note: Physical positions and genes are based on soybean reference genome Glyma. Wm82.a1.v1.1

Supplementary Table 3 The detected SNPs associated with seed protein content using mrMLM procedure in the soybean association panel

SNP	Chromosome	Position	Effect	$-\log_{10} P$	R^2 (%)	R^2 (%) under RTM-GWAS procedure
Gm06_40739891	6	40739891	-0.34	7.2	5.0	-
Gm07_15027859	7	15027859	-0.45	5.4	8.0	-
Gm07_30989289	7	30989289	-0.44	3.9	2.4	-
Gm14_16005146	14	16005146	0.45	3.8	2.7	-
Gm14_32054255	14	32054255	0.43	5.6	5.6	-
**Total (5)**					**23.6**	

Supplementary Table 4 The detected SNPs associated with seed oil content using mrMLM procedure in the soybean association panel

SNP	Chromosome	Position	Effect	$-\log_{10} P$	R^2 (%)	R^2 (%) under RTM-GWAS procedure
Gm05_41503945	5	41503945	0.31	4.6	5.1	-
Gm06_39998760	6	39998760	-0.37	7.5	9.9	-
Gm06_40810272	6	40810272	0.15	5.6	3.7	-
**Gm06_43202237**	6	43202237	-0.22	4.7	2.0	0.6
Gm08_2468904	8	2468904	-0.15	5.0	3.7	-
Gm11_38464734	11	38464734	-0.21	5.0	3.8	-
Gm14_28259042	14	28259042	0.24	5.2	5.8	-
Gm15_16119053	15	16119053	0.16	4.3	4.1	-
Gm20_8373052	20	8373052	0.27	4.4	2.9	-
**Gm20_31299487**	20	31299487	0.41	9.2	7.3	15.1
Total (10)					48.4	

[a] SNPs in bold font type are also detected by RTM-GWAS procedure
